# Supplementary material for: Effect of miR-302b MicroRNA Inhibition on Chicken Primordial Germ Cell Proliferation and Apoptosis Rate
Source: Genes (Basel). 2021 Dec 28;13(1):82. doi: 10.3390/genes13010082 (PMC8774308; doi:10.3390/genes13010082)
Supplement: Supplementary file 1 [file genes-13-00082-s001.zip › genes-1500513-supplementary.pdf]

genes
MDPI

Supplementary Materials

Figure S1: Description of the experiment

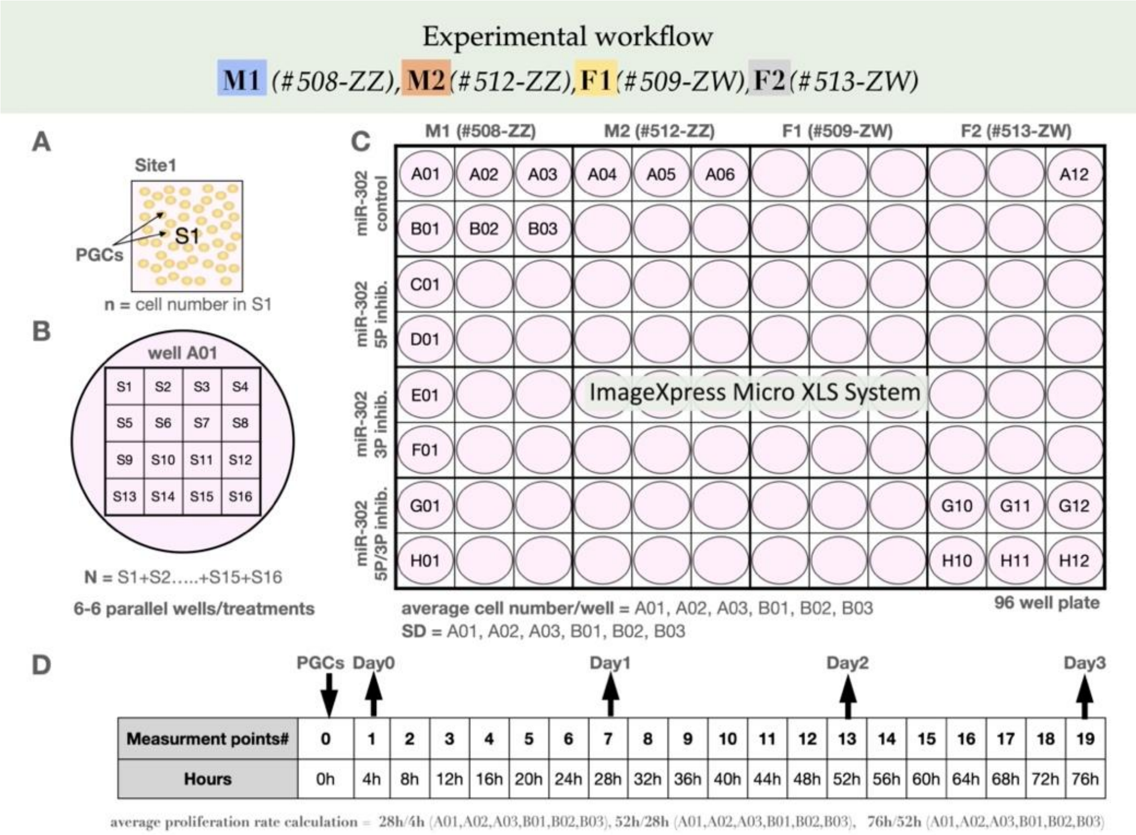

Supplementary Figure S1. There were two male (**M1:** #508-ZZ; **M2:** #512-ZZ) and two female (**F1:** #509-ZW; **F2:** #513-ZW) PG cell lines used in this study. The cell number of these cell lines was measured every 4 hours (h), for three days, using the XLS Imaging system with a built-in incubator. Our aim was to examine the proliferation rate of the PGCs on Day1, Day2, and Day3 after the inhibition of gga-miR-302b-5P (**5P**) or gga-miR-302b-3P (**3P**) or using anti-gga-miR-302b-5P and anti-gga-miR-302b-3P inhibitors combined (**5P/3P inhibition**). E.g., proliferation rate on the third day was calculated by dividing the average cell number counted on the third day (h76) by the second day (h52). We compared the proliferation rate of control and treated lines on the first day (28h/4h), on the second day (52h/28h) and on the third day (76h/52h)

Figure S2: Immunostaining of control M1 PGCs on Day1

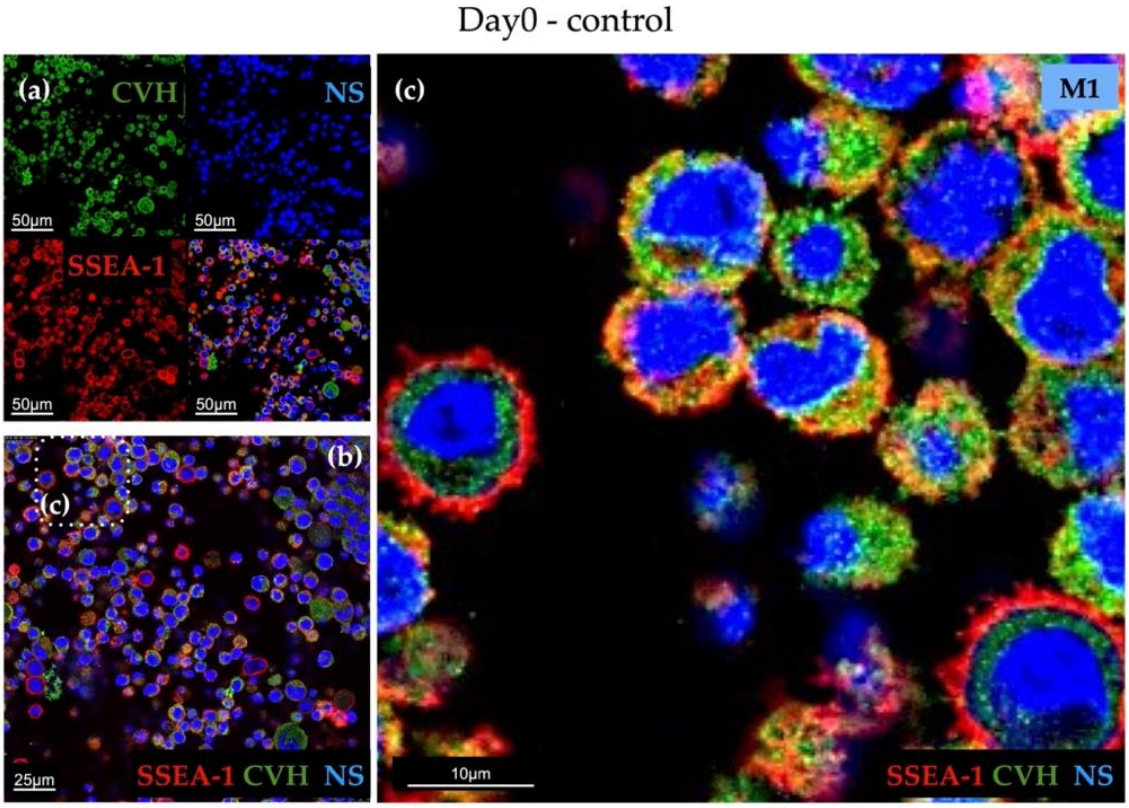

Supplementary Figure S2. Immunostaining of control M1 PGCs on Day1 of cultivation. The immunostaining was done with SSEA-1 (red), CVH (green) and TO-PRO™-3 for nuclear staining (blue). (Scale: **a:** 50 µm **b:** 25 µm **c:** 10 µm).

Figure S3: Immunostaining of control M1 PGCs on Day3

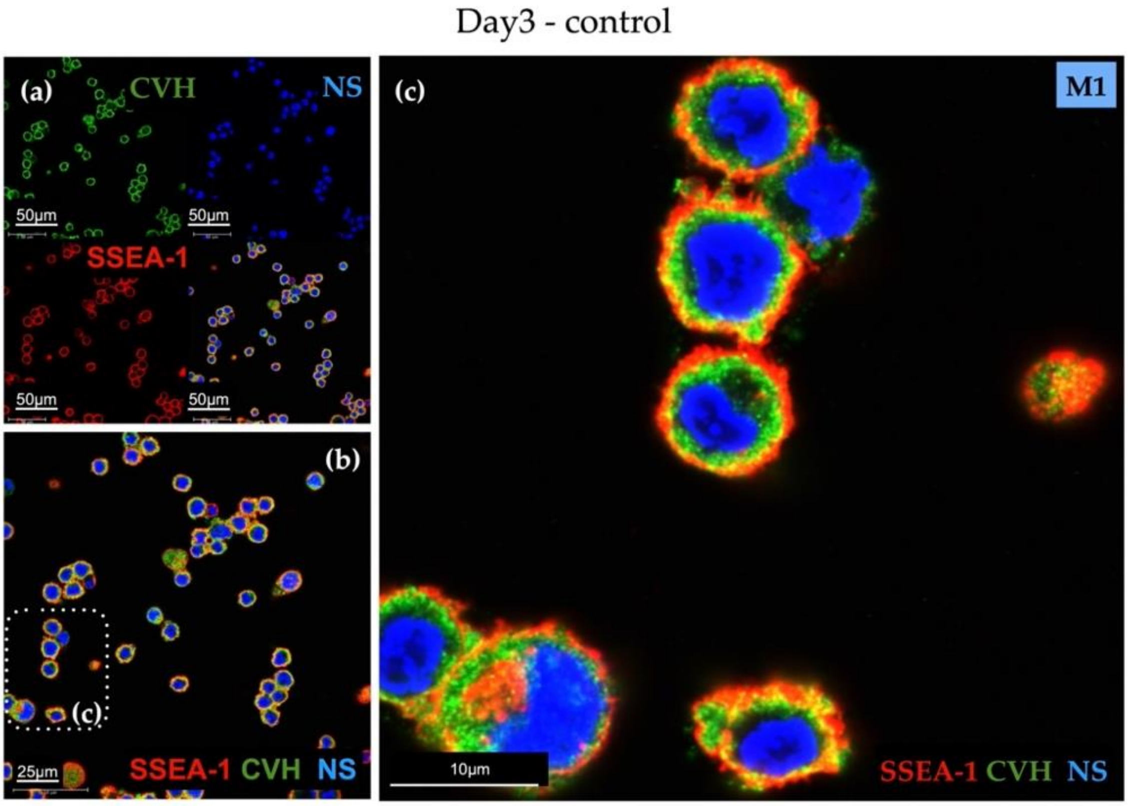

Supplementary Figure S3. Immunostaining of control M1 PGCs on Day3 of cultivation. The immunostaining was done with SSEA-1 (red), CVH (green) and TO-PRO™-3 for nuclear staining (blue). (Scale: **a:** 50 μm **b:** 25 μm **c:** 10 μm).

Figure S4: Immunostaining of 5P inhibited M1 PGCs on Day3

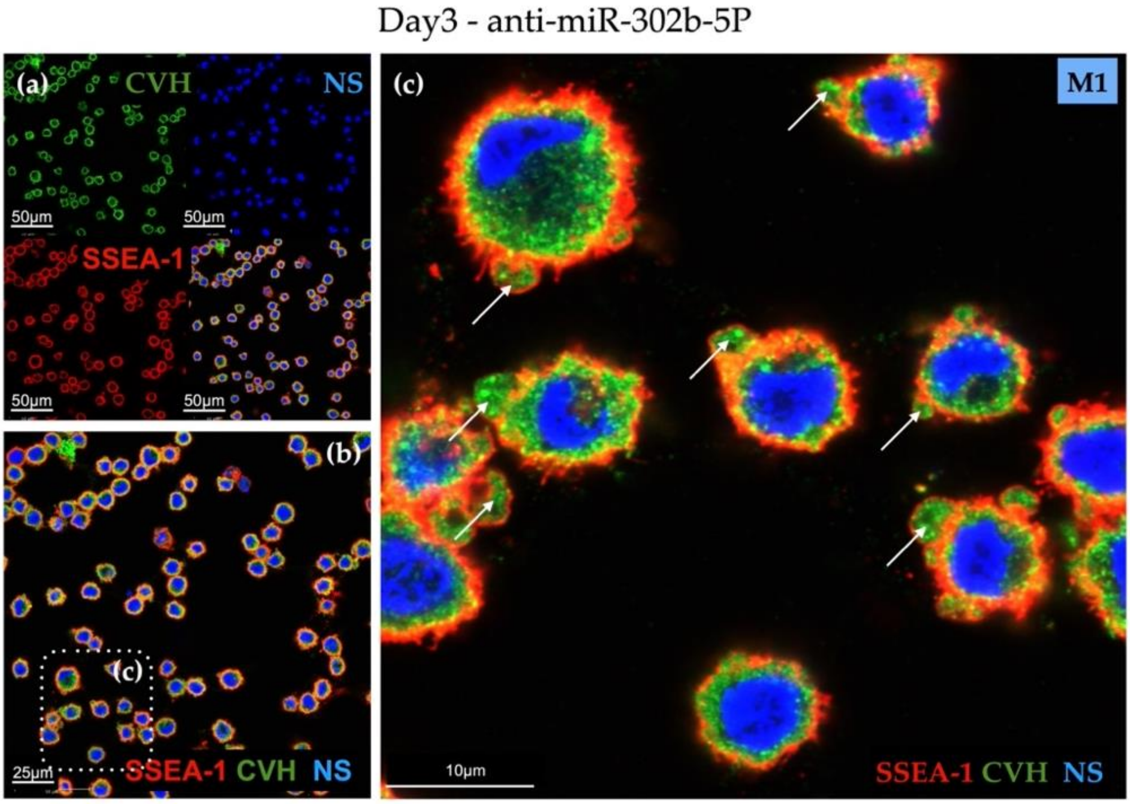

Supplementary Figure S4. Immunostaining of M1 PGCs after the inhibition of gga-miR-302b-5P (5P) on Day3 of cultivation. The immunostaining was done with SSEA-1 (red), CVH (green) and TO-PRO™-3 for nuclear staining (blue). (Scale: **a:** 50 µm **b:** 25 µm **c:** 10 µm).
